# Pathogen-Imprinted Organosiloxane Polymers as Selective Biosensors for the Detection of Targeted *E. coli*

**DOI:** 10.3390/c4020029

**Published:** 2018-05-14

**Authors:** Maria T. Dulay, Naina Zaman, David Jaramillo, Alison C Mody, Richard N Zare

**Affiliations:** Department of Chemistry, Stanford University, Stanford, CA 94305-5080, USA; mdulay@stanford.edu (M.T.D.); naina@gmail.com (N.Z.); dej@berkeley.edu (D.J.); amody1@villanova.edu (A.C.M.)

**Keywords:** organosiloxane, sol–gel, imprinting, biosensor, *E coli*, sensitivity, selectivity ratio, bacteria template

## Abstract

Early detection of pathogens requires methods that are fast, selective, sensitive and affordable. We report the development of a biosensor with high sensitivity and selectivity based on the low-cost preparation of organosiloxane (OSX) polymers imprinted with *E. coli*-GFP (green fluorescent protein). OSX polymers with high optical transparency, no cracking, and no shrinkage were prepared by varying several parameters of the sol–gel reaction. The unique shape and chemical fingerprint of the targeted inactivated *E. coli*-GFP were imprinted into bulk polymers by replication imprinting where the polymer solution was dropcast onto a bacteria template that produced a replica of the bacterial shape and chemistry on the polymer surface upon removal of the template. Capture performances were studied under non-laminar flow conditions with samples containing inactivated *E. coli*-GFP and compared to inactivated *S. typhimurium*-GFP. Capture selectivity ratios are dependent on the type of alkoxysilanes used, the H_2_O:silane molar ratio, and the polymerization temperature. The bacteria concentration in suspension ranged from ~6 × 10^5^ to 1.6 × 10^9^ cells/mL. *E. coli*-imprinted OSX polymers with polyethylene glycol (PEG) differentiated between the targeted bacterium *E. coli*, and non-targeted bacteria *S. typhimurium* and native *E. coli*-GFP, achieving selectivity ratios up to 4.5 times higher than polydimethylsiloxane (PDMS) and OSX polymers without PEG.

## 1. Introduction

Interest in identifying pathogenic microorganisms is motivated by the fact that microbial diseases constitute the major cause of death in many countries [1]. The ubiquity of pathogenic microorganisms, such as bacteria, viruses and parasites, in food, water and blood [2] makes it necessary to have effective testing methods that are rapid, sensitive and accurate. Conventional microbiological testing methods are time-consuming, sometimes taking up to seven days, because they often require an amplification step, and these methods lack the high sensitivity needed to detect low concentrations where even a single pathogenic organism in complex biological environments that may include other non-pathogenic organisms can be an infectious dose [3]. For some of these methods, the high cost required to run and maintain them have limited their broad application, particularly in developing countries.

In response for the need for more rapid and sensitive detection of pathogens, there has been a proliferation of research into the development of biosensors that employ receptors, nucleic acids or antibodies as biological recognition components [4]. Molecular recognition approaches developed for the detection of small molecules or larger biomolecules have been leveraged for use in detecting bacteria. Biosensors based on affinity recognition, where cells are captured, bound or docked to a high affinity ligand, binder or molecularly imprinted polymer, are time-consuming, partly because a response is dependent on the formation of a complex between the target pathogen and the recognition element of the biosensor. Additionally, the binding ability of the affinity ligand is dependent on its stability. Detection techniques, including quartz crystal microbalance, laser-induced fluorescence, electrochemistry and mass spectrometry have been coupled with biological recognition elements [5–10].

In recent years, microbial capture has been realized using target-specific imprinted polymer surfaces for specific and high-affinity capture of pathogenic bacteria. Through molecular imprinting (MIP), biological receptor mimics can be formed within a polymer matrix, allowing for specific recognition of whole cells, like bacteria and viruses [4,11,12]. Unlike molecular recognition elements like antibodies, MIP polymers can be designed to have high physical and chemical stabilities and may be reused multiple times. Dickert and co-workers [13–15] and others [16,17] have developed molecularly imprinted sol–gel polymers using the surface imprinting of template species, which when removed, leave captured in the polymer cavities that are complementary to the template morphology. These cavities act as binding sites for the template, and therefore, are useful in sensing or capturing whole microorganisms at the nanometer and submicron scales. The major advantage of microorganism-imprinted surfaces is the avoidance of any amplification or concentration step required for low cell counts, offering a big advantage in the rapid detection of dilute concentrations of pathogenic bacteria, and possibly including pathogenically viable microorganisms that are non-culturable as a response to environmental stress [18].

Recently, we have built upon the MIP of whole cells by developing a cell-imprinting process for the imprinting of bacteria on polydimethylsiloxane (PDMS) polymers [19–21]. The resulting imprinted PDMS polymers are capable of detecting pathogenic microorganisms in liquids selectively and with high sensitivity because of spatially organized specific recognition cavities formed by imprinting template bacterial cells in a crosslinked PDMS matrix. This imprinting technique, based on nanoimprint lithography [22,23], allows for tailoring three-dimensional micro- and nanostructured surfaces of targeted pathogens onto a surface. By using nanoimprinting lithography, three-dimensional structures with sizes ranging from several micrometers to sub-nanometer scales can be realized [24,25]. Studies utilizing bacteria-imprinted PDMS suggested that the morphology and the chemical fingerprint of the targeted bacteria in the imprint cavities play a role in the re-adsorption of the bacteria [20]. Although PDMS has been shown to be an appropriate polymer for designing imprinted polymer biosensors, it has synthetic limitations. Unlike PDMS, organosiloxane (OSX) polymers made by sol–gel chemistry [26] can be easily prepared with different mechanical and chemical properties by altering different synthetic parameters, namely the choice of silane starting material, catalyst, the molar ratio of water to silane, and polymerization temperature. The OSX polymer can be adapted as required, resulting in polymers with a wide range of functionality leading to enhanced capture sensitivity and selectivity of an imprinted OSX polymer that can “remember” the targeted bacterium.

We describe here the capture performance of *E. coli*-GFP (green fluorescent protein) imprinted OSX polymers for selective and sensitive capture of the targeted rod-shaped, Gram negative bacteria compared to the capture of non-targeted *S. typhimurium*-GFP with similar morphology. These imprinted polymers were characterized by scanning electron microscopy (SEM), and desorption electrospray mass spectrometry (DESI-MS) was used to probe the chemical nature of the imprint cavities for any chemical functionality left behind by the bacteria during imprinting. It has been shown that the mechanism of capture relies both on morphology and chemical recognition of the imprints by the targeted bacteria [20]. The use of sol–gel chemistry to prepare organosiloxane polymers allows us to improve the spatial organization of bacteria-recognition cavities in the polymer, which is important in the selective capture of targeted bacteria.

## 2. Materials and Methods

## 2.1. Materials

Methyltrimethoxysilane (MTMS), dimethyldimethoxysilane (DMDMS), polyethylene glycol MW 10,000 (PEG-10), and 25% aqueous glutaraldehyde solution were purchased from Sigma-Aldrich (St. Louis, MO, USA) and used without further purification. Methanol (analytical grade), PBS 1X (pH 7.4), distilled bio-grade water, and hydrochloric acid were purchased from ThermoFisher Scientific (Waltham, MA, USA). The PDMS kit was purchased from R.S. Hughes (Sunnyvale, CA, USA), which contains a mixture of vinyl-terminated PDMS oligomers, crosslinkers of polysiloxanes with vinyl and hydrogen groups as the major components [27]. *E. coli*-GFP (25922GFP) and *S. typhimurium*-GFP (subspecies *enterica* serovar Typhimurium GFP, 14028GFP) were purchased from ATCC (Manassas, VA, USA). *S. epidermidis*-GFP cultures were obtained from Niaz Banaei (Medical Center, School of Medicine, Stanford University, Stanford, CA, USA).

Polystyrene multiculture plate lids (6-well, Beckton-Dickinson) were purchased from ThermoFisher Scientific (Waltham, MA, USA) without washing or pretreatment. Thermanox coverslips (10.5 × 22 mm, 26025) were purchased from Ted Pella (Redding, CA, USA).

## 2.2. Cell Handling

Bacterial cells were streaked on Luria broth (LB) agar plates and grown for 24 h at 37 °C in an incubator. To prepare suspensions of bacterium for template preparation and cell-capture experiments, freshly grown bacterial cells were harvested from culture and inoculated into 2–4 mL phosphate-buffered saline (PBS) 1X buffer and vortexed for 30 s to suspend the cells. Washing the suspension involved (1) centrifuging at 5000 rpm for 5 min at 4 °C, (2) resuspending of the cell pellet in 1 mL volume of PBS 1X or distilled bio-grade water after removal of the supernatant, (3) vortexing for 30 min, and (4) centrifuging at 2000 rpm for 3 min at 4 °C. The washing protocol was repeated 1 more time before finally resuspending the cell pelleting in 0.5–1 mL PBS 1X or distilled bio-grade water before measuring bacterial cell density at OD_600_. The stock suspension was prepared with OD_600_ of approximately 2.

Thermal inactivation of bacterial cells was achieved by heating at 80 °C for 1 h followed by gentle rinsing with 1 mL distilled bio-grade water. Chemical inactivation was performed by adding glutaraldehyde solution to the suspension until the final glutaraldehyde concentration was 2.5% (*v*/*v*) and allowed to react at room temperature for 1.5 h. The glutaraldehyde-inactivated bacterial cells were washed by centrifugation at 2000 rpm for 5 min at 4 °C then resuspended in distilled bio-grade water and vortexed for 30 s. This wash step was repeated. The final suspension was prepared with an OD_600_ between 1 and 2. Chemical inactivation was confirmed by streaking the suspension on an LB agar plate and incubated at 37 °C for 24 h, after which no colonies were observed. The final suspension was used to prepare aliquots of different OD_600_ values and stored at 4 °C for up to 1 year. Bacterial cells were excited with a 488 nm blue laser and visualized at its emission at 520 nm.

## 2.3. Template Preparation

Volumes between 1 and 3 µL of native bacterial suspension/aliquots of different OD_600_ values were deposited on the surface of a polystyrene substrate and heated at 80 °C for 1 h. Templates were prepared with concentrations of bacteria in suspensions, ranging from 3.2 × 10^8^ (OD_600_ 0.4) to 1.6 × 10^9^ (OD_600_ 2) cells/mL. Similarly, 1–3 µL of glutaraldehyde-inactivated bacterial suspension were deposited on the hydrophobic side of a Thermanox coverslip, then placed at 4 °C for 24 h. The droplets were evaporated to dryness at ambient conditions for 1–2 h before gentle rinsing with 1 mL of distilled bio-grade water. Dry templates were stored covered and in the dark at room temperature when not used. Templates were reused 5 times each without the need for rinsing or washing between uses.

## 2.4. Preparation of OSX and Polydimethylsiloxane (PDMS) Polymers

Four different OSX polymers were prepared as shown in Table 1 OSX-A polymer was prepared by mixing 300 µL 1:2 (*v*/*v*) DMDMS to MTMS and 50 µL 0.12 M HCl, then stirred at room temperature for 2.5 h, forming a clear reaction solution. Heating at 65 °C for 24–30 h resulted in the formation of a polymer. OSX-B polymer was prepared by mixing 300 µL 1:2 (*v*/*v*) DMDMS to MTMS in 50 µL 0.012 M HCl, then stirred at room temperature for 2.5 h. Polymerization occurred at 65 °C for 16–18 h. OSX-C and OSX-D were prepared by adding 300 µL MTMS and 10% (*w*/*v*) PEG-10 in 0.12 M HCl with a R of 1.32 and 1.83, respectively. The reaction solutions were stirred at room temperature (25 °C) for 2.5 h and polymerized at 25 °C for 24 h. After polymerization, imprinted polymers were washed with a gentle stream of 1 mL volume of distilled bio-grade water followed by sonication for 5 min in water.

**Table 1:** Reaction conditions in synthesis of organosiloxane (OSX) polymers used for imprinting

Polymer	Silane(s)	[HCl] (M)	Additive	Reaction T (◦C)	H_2_O:Si Molar Ratio
OSX-A	MTMS:DMDMS (2:1, *v*/*v*)	0.12	None	65	1.32
OSX-B	MTMS:DMDMS (2:1, *v*/*v*)	0.012	None	65	1.32
OSX-C	MTMS	0.12	PEG-10	25	1.32
OSX-D	MTMS	0.12	PEG-10	25	1.83

PDMS polymer solution was prepared by mixing 10 parts monomer and 1 part crosslinker, followed by degassing in a dessicator at low-pressure. The polymer solution was cured at 80 °C for 1 h.

## 2.5. Imprinting of OSX and PDMS Polymers

**Scheme 1:**
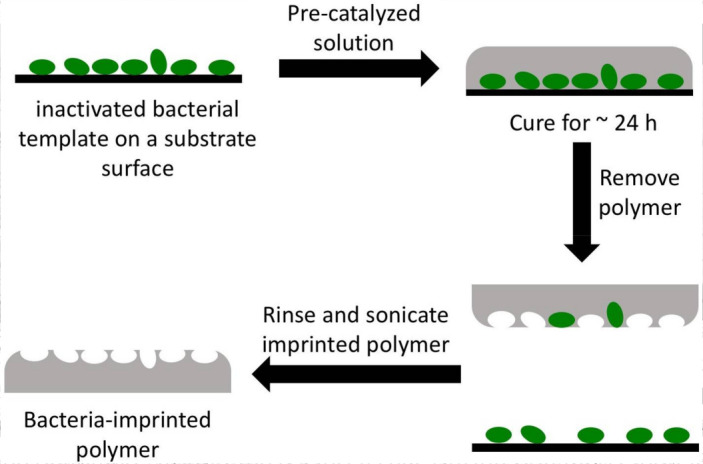
Replication imprinting workflow. Green objects represent bacteria adhered to a polystyrene or hydrophobic Thermanox substrate surface.

Scheme 1 illustrates the process flow of imprinting polymers with bacterial cells. A volume of 100–200 µL of polymer reaction solution (after 2.5 h stirring at 25 °C) was deposited directly on top of a bacterial template placed in a container that was loosely covered during polymerization. The solution polymerized at approx. 25 °C or 65 °C. The imprinted polymer was peeled from the template surface, washed with 0.5–1 mL distilled bio-grade water, then sonicated for 5 min while immersed in water to remove any adhered bacterial cells and allowed to dry under ambient conditions before use. Imprinted polymer samples were coated with Au/Pd using a Denton Desk II sputtering unit before imaging with a Hitachi S-3400N VP-SEM (Schaumburg, IL, USA). Imprinted polymers were imaged with a confocal fluorescent microscope (TCS SP2, Leica, Wetzlar, Germany) to ensure that all bacterial cells had been removed from the surface before use in capture experiments. Imprinted polymers were stored in sealed containers and kept in the dark at room temperature.

The selectivity ratios of imprinted polymers toward the capture of its targeted bacteria were calculated using the following equation:

$$\text{selectivity ratio}=\frac{\text{mean \# of captured E. coli per view field}}{\text{mean \# of other microorganisms captured}}$$

## 2.6. Bacterial Cell Capture

An imprinted OSX polymer was adhered to the bottom of a small Petri dish using small piece of double-sided adhesive tape. Approximately 3 mL of bacterial suspension was added to the Petri dish, ensuring that the imprinted polymer was completely immersed in the suspension. The covered Petri dish was placed on a shaker in a 37 °C room for 30 min. The imprinted polymer was removed from the suspension and gently rinsed with a stream of distilled bio-grade water (~0.5 mL), then dried under ambient conditions before visualization. The imprinted area of the polymer was inspected under a confocal microscope (TCS SP2, Leica) equipped with an argon-ion laser for excitation of the green fluorescent protein at 488 nm. Emission data was collected at 520 nm and processed using Image J software (open source Java image processing program, http://imagej.net/ImageJ).

## 2.7. Desorption Electrospray Ionization Mass Spectrometry (DESI-MS) Analysis

The chemical profile of glutaraldehyde-inactivated *E. coli*-GFP templates and imprinted OSX polymers were studied with DESI-MS. We utilized a lab-built DESI-MS source coupled to an LTQ-Orbitrap XL mass spectrometer (Thermo Scientific, San Jose, CA, USA). DESI-MS was performed in the negative ion mode from *m*/*z* 50–1000 using an orbitrap as the mass analyzer. The spray solvent 9:1 (*v*/*v*) methanol:H_2_O was used for analysis at a flow rate of 5 µL/min; the N_2_ pressure was set to 150 psi for the nebulizing gas, and the spray voltage was set to 5 kV.

## 3. Results and Discussion

## 3.1. Preparation of Organosiloxane (OSX) Polymers

The appropriate OSX polymer requires easy template removal and efficient integration into a fluorescence-based sensing approach where optical transparency is important. Four different OSX polymers (Table 1) were prepared using sol–gel chemistry to create imprinted polymers with high optical transparency and mechanical robustness with no lateral shrinkage and no cracking. Sol–gel chemistry involves the preparation of a sol (colloidal solids suspended in a solution), gelation of the sol, then formation of a monolith via evaporative drying [26]. Many studies have shown that variations of sol–gel synthesis conditions cause modifications in the physicochemical properties of the polymer, such as degree of crosslinking, pore distribution and hardness [28,29]. These variations influence the kinetics of the hydrolysis and condensation reactions involved in sol–gel chemistry; these rates are the most important factors that influence the final polymeric structure [30]. Several reaction parameters known to influence the polymer structure were varied: types of silane reagents, such as methyltrimethoxysilane (MTMS) and dimethyldimethoxysilane (DMDMS) used in this study, temperature, the molar ratio (R) of H_2_O:silane, acid catalyst concentrations, and addition of polyethylene glycol with MW 10,000 (polyethylene glycol, PEG-10). The use of methyl-substituted alkoxysilanes, such as MTMS and DMDMS, aid in minimizing or avoiding cracking during evaporative drying of the polymer by allowing for structural relaxation through a spring-back effect by the methyl groups within the cavities of the polymer network [31]. The presence of cracking leads to deformations of the imprint cavities that may affect the capture performance of the OSX polymers. OSX-A and OSX-B polymers with high optical transparency, little to no cracking, and high mechanical stability were produced when R was 1.32, in the absence of PEG-10 at 65 °C and 0.12 N HCl and 0.012 N HCl, respectively, as expected when the sol–gel reaction conditions favor the condensation reactions at R < 2 [28]. The type of acid catalyst has been reported to have very little effect on the polymer structure [28], but we observed that HCl was a better catalyst than acetic acid, which often resulted in polymers with lateral shrinkage. Clear and transparent polymers are obtained when the concentration of HCl is above 0.008 N [28], which we observed to be the case for OSX-A and OSX-B. There was no pronounced difference in the appearance of polymers prepared at the lower acid catalyst concentration. However, polymerization time was reduced significantly from 24–30 h for 0.012 N HCl to 16–18 h for 0.12 N HCl. Therefore, 0.12 N HCl was used in subsequent preparations of OSX polymers. Although MTMS and DMDMS have been reported to eliminate cracking in the polymers, cracking was observed in some of the OSX polymers prepared. It is thought that the stress put upon the polymer as it equilibrates from the high reaction temperature to room temperature may account for the cracks [32]. To reduce the possibility of cracks forming in the polymers, the temperature was decreased to 25 °C (room temperature). At this temperature, however, complete polymerization required more than 3 days.

To shorten the polymerization time at 25 °C, PEG-10 was added to the reaction mixture. Numerous studies have shown PEG to be a structure-directing agent in sol–gel reactions [28]. The addition of a structure-directing agent, such as PEG-10, increases the rate of sol–gel polymerization through self-assembly between PEG molecules and the growing sol–gel oligomers, resulting in shorter curing times [33] Polymers OSX-C and OSX-D, which possessed similar physical properties, were obtained at R 1.32 and 1.83, respectively, in the presence of PEG-10 at 25 °C. Complete polymerization was achieved within 16–18 h. At the lower polymerization temperature, cracking was avoided in all polymers synthesized. Increasing the R values led to faster hydrolysis rates and slower condensation rates [28]. At R > 4, the OSX polymers were not transparent and prone to cracking or incomplete polymerization, leading to undesirable polymers for imprinting. In the presence of PEG-10, OSX-C and OSX-D were optically transparent, free of cracks, and slightly flexible with high mechanical stability. The presence of PEG-10 allowed polymerization to proceed at room temperature (25 °C), simplifying the synthesis of OSX polymers and avoiding any possible morphological changes to the bacterial cells in the template that can occur at higher temperatures. We also note that our OSX polymers are stable when exposed to phosphate and citrate buffers.

## 3.2. Capture Selectivity and Sensitivity of Imprinted OSX Polymers

Two closely related bacteria, *E. coli* and *S. typhimurium*, were chosen to investigate the ability of imprinted OSX polymers to discriminate between the two bacteria using fluorescence imaging. Both *E. coli* and *S. typhimurium* are Gram negative with rod-shaped morphology (about 2 µm by 0.5 µm) [34]. Three different methods of imprinting *E. coli* onto OSX polymers were evaluated. Stamp imprinting, where the bacterial cell template is pressed on top of the polymer, did not reproducibly generate imprints of the cells because the polymer was not of adequate viscosity for imprinting. Partial curing of bulk OSX polymers (>4 mm thickness) to achieve an appropriately viscous substance was not always reliably achieved. Mixing of the bacterial cells into the polymer solution resulted in very little imprint cavities on the surface. Most of the bacterial cells remained within the polymer network and were not accessible during washing and, therefore, were not removed. The preferred imprinting method is analogous to replication imprinting [35] where the first step involved the deposition of a non-viscous sol–gel polymer solution over the bacterial template followed by curing (see Scheme 1). The hydrophobic nature of the template substrate helped to prevent spreading of the mostly aqueous polymer reaction solution, allowing good control over the alignment of the polymer droplet and the bacterial cells of the template, resulting in an OSX polymer with the imprint area positioned at the center of the OSX polymer piece.

We studied the recognition performances of OSX-A, OSX-B, OSX-C, OSX-D imprinted with heat-fixed or glutaraldehyde-inactivated *E. coli*-GFP bacteria. Table 2 lists the selectivity ratio and the sensitivity (the number of cells captured) of each polymer. The selectivity ratios indicate preferential binding of targeted inactivated bacteria over binding of inactivated non-targeted *S. typhimurium*. Capture of targeted and non-targeted bacteria proceeded under non-laminar flow conditions and did not require continuous flow of the bacteria sample solution during the capture period.

**Table 2:** Capture of targeted inactivated *E. coli*-GFP bacteria on OSX and polydimethylsiloxane (PDMS) polymers imprinted with the same inactivated bacterium. Selectivity ratios based on capture of non-targeted inactivated *S. typhimurium*. Total captured is based on number of targeted or non-targeted bacterial cells in 10 view fields. Selectivity was determined with *S. typhimurium* as the non-targeted bacterium (*n* = 2).

Polymer	Imprint Bacteria	Total *E. coli* Captured	Total *S. typhimurium* Captured	Selectivity Ratio
OSX-A	*E. coli (heat fixed)*	120 ± 40	17 ± 4	7.1 ± 1.5
OSX-B	*E. coli (heat fixed)*	108 ± 20	38 ± 10	2.8 ± 2.0
OSX-C	*E. coli (glutaraldehyde inactivated)*	136 ± 25	5 ± 1	27.2 ± 5.2
OSX-D	*E. coli (glutaraldehyde inactivated)*	281 ± 42	26 ± 11	10.8 ± 7.4
PDMS	*E. coli (glutaraldehyde inactivated)*	80 ± 13	8 ± 2	10.0 ± 4.2

**Figure 1:**
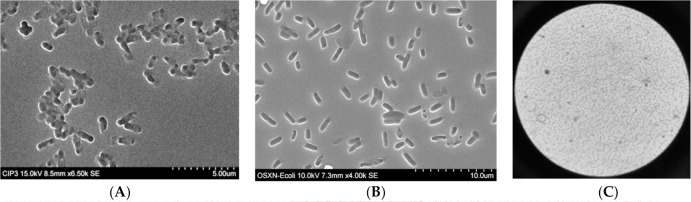
Scanning electron microscopy (SEM) images of (**A**) imprinted OSX-B polymer prepared with heat-fixed *E. coli*-GFP (OD_600_ 1.0 suspension) on polystyrene substrate, (**B**) glutaraldehyde-inactivated *E. coli*-GFP on OSX-C polymer prepared with *E. coli*-GFP (OD_600_ 0.44 suspension) on Thermanox, and (**C**) bright-field image of glutaraldehyde-inactivated *E. coli*-GFP template at 40X magnification.

The imprinted OSX-A polymer captured a higher number (120 ± 40) of the targeted bacterium compared to the capture of non-targeted heat-fixed *S. typhimurium*-GFP (17 ± 4), resulting in a capture selectivity ratio of 7.1 ± 1.5. Heat-fixed *E. coli*-GFP-imprinted OSX-B polymer demonstrated similar selectivity ratio (2.8 ± 2.0) as OSX-A for the targeted bacteria, indicating that the higher HCl catalyst concentration did not significantly enhance the ability of the imprinted OSX-A polymer to differentiate between *E. coli* and *S. typhimurium*. Imprinted OSX-B exhibited lower sensitivity, capturing 108 ± 20 bacterial cells over the same number of view fields as OSX-A. SEM images in Figure 1A show that the rod shape of heat-fixed *E. coli*-GFP with average length of 1.7 µm was captured at the surface of OSX-B polymer during its imprinting. Throughout the imprint area, whose size is defined by the template area (3–4 mm in diameter), are clusters of and isolated rod-shaped imprint cavities, a replication of the arrangement of bacterial cells on the template.

Heat-fixed bacteria imprinted OSX polymers were also prepared with two other alkoxysilane reagents, diethoxydimethylsilane and aminopropyldimethoxysilane. When used in combination with methyldimethoxysilane and prolonged polymerization at 65 °C, diethoxydimethylsilane did not produce a polymer even after 1 week. Aminopropyldimethoxymethylsilane produced opaque polymers not suitable for fluorescent detection of bacteria captured on imprinted polymers.

OSX-C and OSX-D were imprinted with templates of glutaraldehyde-inactivated bacteria adhered to hydrophobic Thermanox coverslips. Both imprinted polymers were prepared at 25 °C with MTMS only in the presence of polyethylene glycol (PEG-10) as a structure-directing agent and a passivating agent to reduce non-specific adsorption of bacteria onto the non-imprinted areas of the OSX polymer. The SEM image in Figure 1B shows the arrangement of glutaraldehyde-inactivated *E. coli*-GFP imprint cavities on OSX-C. The rod-shaped cavities have an average length of 2.5 µm, which contrast the shorter rods of heat-activated *E. coli*-GFP (Figure 1A). SEM analysis of imprinted OSX-D revealed a similar imprinted surface as OSX-C. Figure 1C is a magnified bright-field view of the bacterial cells in the template. The size of the template area is between 3 mm and 5 mm, which is entirely replicated on the OSX surface during polymerization. The coffee-ring of bacteria, containing the highest density of cells at the circumference of a circular bacterial template and formed during the preparation of the template, is clearly replicated in the polymer (not shown). Chemical inactivation was the preferred method of inactivating the bacteria to avoid the possible aerosolization of live bacteria during the heat-fixation process.

**Figure 2:**
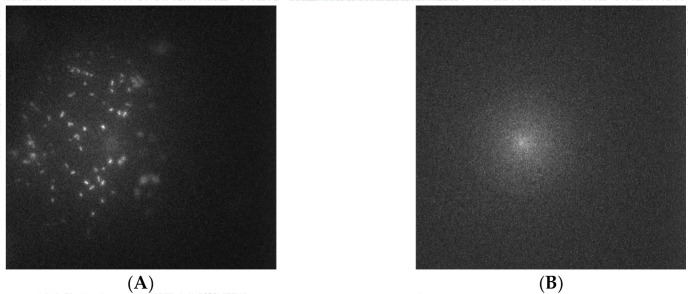
Fluorescent images of captured (**A**) glutaraldehyde-inactivated *E. coli*-GFP (OD_600_ 0.38 capture solution) and (**B**) glutaraldehyde-inactivated *S. typhimurium*-GFP (OD_600_ 0.38 capture solution) on a glutaraldehyde-inactivated *E. coli*-GFP-imprinted OSX-C polymer.

Captured glutaraldehyde-inactivated *E. coli*-GFP from an OD_600_ 0.38 suspension on glutaraldehyde-inactivated *E. coli*-GFP-imprinted OSX-C appeared as bright white “dots” in the fluorescence image as shown in Figure 2A. In contrast, the same imprinted OSX-C polymer did not show any capture of non-targeted glutaraldehyde-inactivated *S. typhimurium* from an OD_600_ 0.38 suspension as shown in Figure 2B, revealing high affinity of OSX-C towards *E. coli* cells.

Changing the reaction parameter, R, affected both the sensitivity and selectivity of capture for the targeted glutaraldehyde-inactivated *E. coli*-GFP for OSX-C and OSX-D as shown in Table 2. The trend in Table 2 seems to indicate that increasing R leads to high sensitivity with low selectivity. The selectivity ratio increased nearly 2.5 times when the R was decreased: an OSX-C selectivity ratio of 27.2 with R of 1.32 was compared to 10.8 for OSX-D with R of 1.83. In contrast, capture sensitivity increased nearly 2 times when R was increased: OSX-D captured 281 ± 22 bacterial cells while OSX-C captured 136 ± 15 bacterial cells. Increasing the molar ratio leads to a decrease in the condensation reaction rate, leading to a looser arrangement of the polymer network. This might suggest that the reorganization of the oligomers around the bacterial cells during polymerization is more efficient at a higher R value, resulting in more imprint cavities available for capture at the OSX-D surface. The reason for the increase in selectivity ratio when R is decreased is not well understood. When compared to imprinted PDMS, both polymers provided higher selectivity ratios. Imprinted OSX-C and OSX-D were 1.7 and 3.5 times more sensitive than PDMS in capturing targeted *E. coli*, respectively.

Glutaraldehyde-inactivated *E. coli*-GFPimprinted OSX-C polymer exhibited nearly 10 times higher selectivity ratio (27.2) than heat-fixed *E. coli*-GFP-imprinted OSX-B (2.8) polymers for their targeted *E. coli*-GFP, as shown in Table 2. Both polymers were prepared with R 1.32; OSX-C was prepared at 25 °C and OSX-B at 65 °C (Table 1). The slower polymerization process and the use of a single hydrophobic silane (MTMS) involved in creating imprinted OSX-C may allow for better reorganization of the intermediate polymer strands around the bacterial cells in the template, capturing better the morphology and chemistry of the bacteria when compared to OSX-B.

All four OSX polymers had more pronounced sensitivity to the targeted bacteria than PDMS, as shown in Table 2. OSX-A and OSX-B, both prepared in the absence of PEG-10 and at high temperature, captured 33% and 26%, respectively, more targeted bacterial cells than PDMS. In contrast, the polymers prepared with PEG-10 and at lower temperature had higher affinities for the targeted bacteria with OSX-D being more pronounced at 71% captured cells than OSX-C at 41%. Imprinted OSX-C was 65% more selective than PDMS in capturing targeted *E. coli* while OSX-D was only slightly more selective. Both OSX-A and OSX-B exhibited only slightly higher selectivity ratios of up to 38% compared to PDMS.

**Figure 3:**
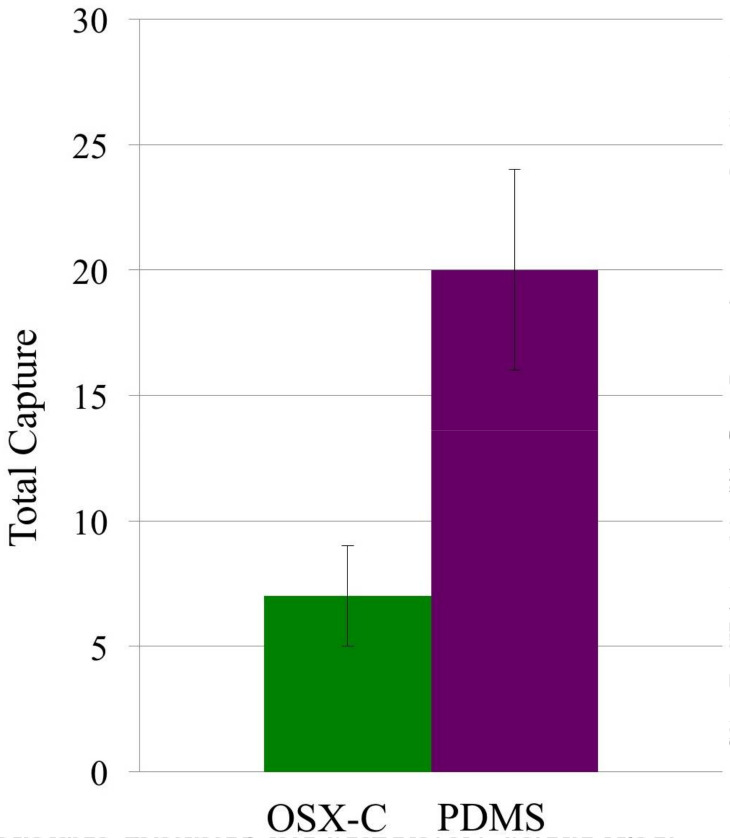
Capture of live *E. coli*-GFP (OD_600_ 0.38 suspension) on a glutaraldehyde-inactivated *E. coli*-GFP-imprinted OSX-C polymer (R = 1.32; cells captured 7 ± 2; green bar) and PDMS (cells captured 20 ± 4; purple bar), *n* = 2.

Capture of native (or live) *E. coli*-GFP was done to further illustrate the selectivity of glutaraldehyde-inactivated *E. coli*-GFP-imprinted OSX-C towards it targeted bacterium. The results can be compared with PDMS imprinted with glutaraldehyde-inactivated *E. coli*-GFP, using the similar bacterial template used for imprinting of the OSX-C polymer. Figure 3 shows that imprinted OSX-C captured less (7 ± 2) of the non-targeted native bacterial cells than imprinted-PDMS (20 ± 4), resulting in a selectivity ratio of 2.9 ± 1.4. Furthermore, the selectivity ratio of 7 ± 2 for OSX-C polymer in the capture of non-targeted native *E. coli*-GFP is approximately 77% lower than the selectivity ratio obtained for the capture of targeted glutaraldehyde-inactivated *E. coli*-GFP (Table 2). Inactivation of the *E. coli*-GFP by glutaraldehyde alters the morphology and presumably the chemistry of the bacterial

wall so that the inactivated bacteria are physically and chemically different from the native bacteria. A study by Chao [36] reports that the effects of chemical inactivation by glutaraldehyde changes the morphology by reducing its length and the density of surface ultrastructures of *E. coli*. Our imprinting process works well for capturing the unique morphology and cell-wall properties of *E. coli* regardless of how it is inactivated, as shown in the SEM images in Figure 1.

**Figure 4:**
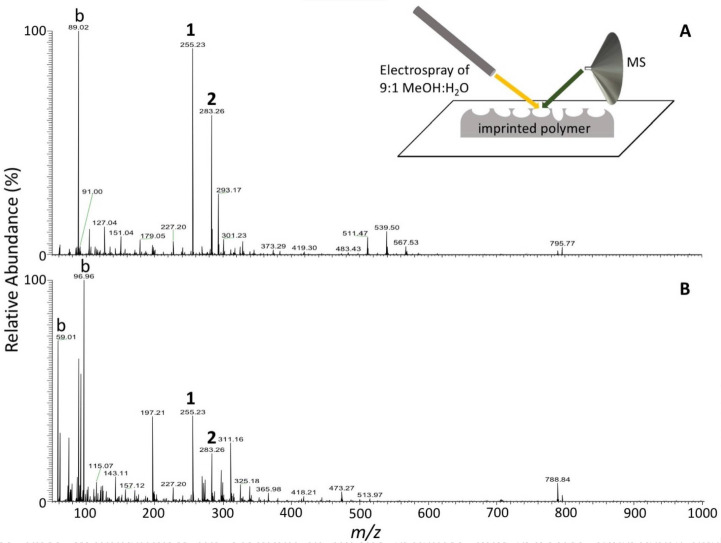
Negative ion mode desorption electrospray (DESI) mass spectra obtained from (**A**) imprinted OSX-C polymer (unused) and (**B**) glutaraldehyde-inactivated *E. coli*-GFP on Thermanox (template). **1**: palmitic acid, **2**: stearic acid, b: background. Inset: schematic diagram of DESI-MS setup.

It is hypothesized that the capture comes from shape, but this is not completely the explanation. In a study by Ren and Zare [20], a monolayer overcoating was applied to the templated PDMS film and the capture capability was effectively removed. Therefore, some form of biorecognition is involved although the nature of this recognition process is not yet fully established. Some of it may come from the prepolymer “feeling” the surface of the microorganism and adjusting to minimize the interaction energy. Some of it may also come from extracellular components deposited in the mold by the imprinted microogranisms. Preliminary studies using DESI-MS to determine the chemical composition of the imprint cavities show a similarity in the mass spectrum of a glutaraldehyde-inactivated *E. coli*-GFP-imprinted OSX-C polymer to that of the bacterial template. In Figure 4A, palmitic acid (peak 1) and stearic acid (peak 2) are observed. These two peaks are also seen in the spectrum of the bacterial cells in the template (Figure 4B). These peaks are not observed from the non-imprinted areas of the polymer or from a polymer that has not undergone any imprinting. It is believed that during imprinting, the chemistry of the bacteria is imprinted along with the morphology. DESI-MS has been used successfully to obtain the chemical signatures of bacteria [37], but its use to obtain direction chemical information from imprint cavities has not previously been reported. Further studies are needed to better understand the chemical nature of the imprint cavities.

## 3.3. Non-Specific Adsorption of Targeted Bacteria on Non-Imprinted Areas of Imprinted OSX Polymers

Non-specifically adsorbed targeted bacteria in the non-imprinted areas of OSX-A and OSX-B were observed to be 7 ± 1 *E. coli*-GFP and 5 ± 2, respectively. However, when PEG-10 was present in the imprinted OSX polymers, OSX-C and OSX-D, no targeted bacterial cells were observed in non-imprinted areas. PEG macromolecules are known to passivate surfaces coated with PEG, preventing bacterial adhesion.

Sol–gel studies have suggested that the association of PEG macromolecules to form densely tangled sol–gel particle-PEG complexes occur only at the surface of the sol–gel polymer, resulting in very low amounts of PEG within the micropores of the sol–gel polymer [30,38]. PEG macromolecules have been shown to prevent bacterial adhesion when surfaces are coated with PEG [39]. In our study, PEG-10 serves two purposes: (1) to reduce the curing time of the polymers, and (2) to prevent or reduce non-specific adsorption of targeted bacteria. Furthermore, non-specific adsorption of bacteria in the non-imprinted areas of the imprinted OSX-C polymer was reduced from the presence of PEG, which plays a role in passivating the polymer surface and, therefore, increasing the selectivity of the polymer for its targeted bacteria.

## 3.4. Detection of Targeted Bacteria at Low Density

**Figure 5:**
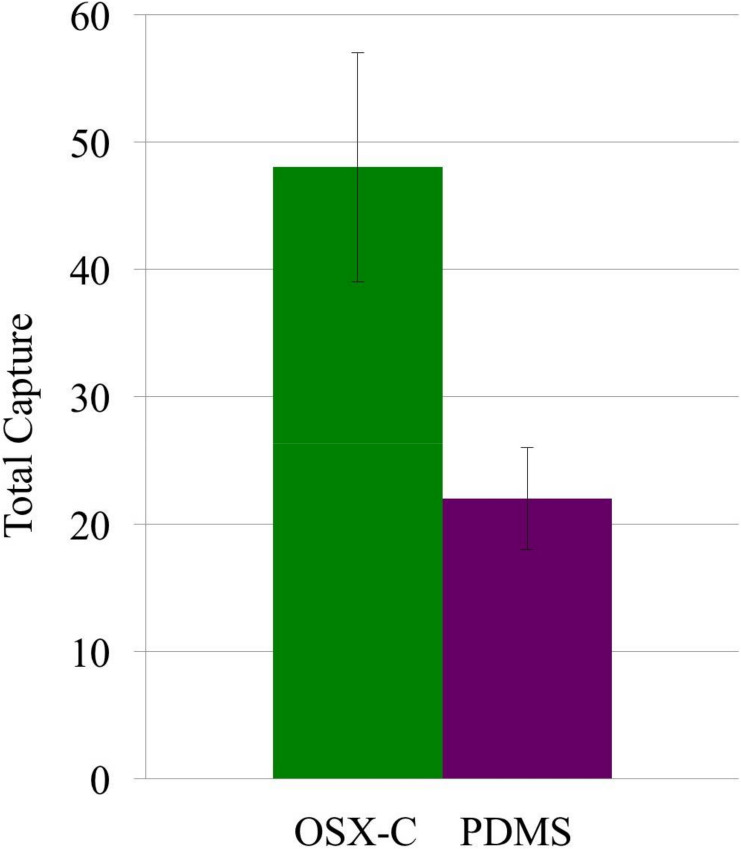
Capture of targeted glutaraldehyde-inactivated *E. coli*-GFP (~6 × 10^5^ cells/mL) on imprinted inactivated *E. coli*-GFP OSX-C polymer (cells captured 49 ± 9; green bar) and PDMS (22 ± 4), *n* = 1. Imprinting was done with template prepared from a glutaraldehyde-inactivated bacterial suspension (OD_600_ 2.0).

OSX-C detected 49 ± 9 cells from a suspension with low *E. coli* density of ~6 × 10^5^ cells/mL, a clinically important concentration in the detection of urinary tract infections [40]. OSX-C was found to be 2.2 times more sensitive than PDMS, which captured 22 ± 4 cells (Figure 5). Imprinted OSX polymers show promise for their use in detecting low densities of bacteria in liquid samples.

## Conclusions

We have demonstrated the creation of highly sensitive and selective biosensors based on replication imprinting of bacterial cells on the surface of OSX polymers prepared by sol–gel chemistry. The results demonstrate that through re-adsorption of targeted bacteria, the imprinted OSX polymers were able to differentiate between targeted and non-targeted bacteria of similar strains with high selectivity ratios. Furthermore, imprinted OSX polymers achieved up to 4.5 times better selectivity than analogous imprinted PDMS polymers. The capture of targeted bacteria involved both shape and chemical recognition. The ease and low-cost of preparation of imprinted OSX polymers offers an alternative to imprinted PDMS polymers and opens new possibilities for the use of these imprinted OSX polymers for rapid and accurate diagnosis of pathogens using a variety of detection techniques that can help to further enhance the selectivity of capture.
